# Overexpression of Tn antigen induces chronic pancreatitis in mice

**DOI:** 10.1038/s41598-025-96060-0

**Published:** 2025-04-02

**Authors:** Baris Mercanoglu, Nina Schraps, Anastasios D. Giannou, Elena Neuburg, Jan Kempski, Christoph Wagener, Nathaniel Melling, Maximilian Bockhorn, Thilo Hackert, Gerrit Wolters-Eisfeld

**Affiliations:** 1 Department of General, Visceral and Thoracic Surgery, University Medical Center Hamburg-Eppendorf, 20246 Hamburg, Germany; 2 Section of Molecular Immunology und Gastroenterology, I. Department of Medicine, University Medical Center Hamburg-Eppendorf, 20246 Hamburg, Germany; 3 Medical Faculty, Universität Hamburg, 20246 Hamburg, Germany; 4 Department of General and Visceral Surgery, University Medical Center Oldenburg, 26133 Oldenburg, Germany

**Keywords:** Pancreatitis, Tn antigen, Cosmc, GalNT2, *O*-Glycosylation, Mouse model, Pancreatitis, Experimental models of disease, Glycobiology

## Abstract

Altered *O*-glycosylation is a key contributor to various pathophysiological processes. Notably, the expression of the Tn antigen is primarily attributed to dysfunction of the chaperone Cosmc, while the overexpression of polypeptide *N*-acetylgalactosaminyltransferases (GalNAc-Ts) has also been implicated in numerous diseases. We generated a transgenic mouse model with conditional *Cosmc-*knockout and simultaneous overexpression of polypeptide *N*-acetylgalactosaminyltransferase 2 (GalNT2) mediated by the *pancreas-specific transcription factor 1a* (*Ptf1a*)-Cre mouse strain to investigate the effect of Tn antigen overexpression on the pancreas in vivo. Histopathological examination of the transgenic pancreas revealed a chronic pancreatitis phenotype with interlobular fibrosis and focal necrosis after only a few weeks as a result of Tn antigen overexpression. In the later stages, there was a progressive loss of pancreatic parenchyma with consecutive exocrine pancreatic insufficiency and malnutrition in the transgenic mice. Flow cytometric analyses have also confirmed that significant infiltration of immune cells occurs in the course of pancreatitis. In the transgenic mouse model presented here, we demonstrated that overexpression of the Tn antigen in the pancreas results in chronic pancreatitis, highlighting the pathophysiological importance of truncated *O*-glycosylation.

**Supplementary Information:**

The online version contains supplementary material available at 10.1038/s41598-025-96060-0.

## Introduction

Glycosylation is one of the most common and diverse forms of posttranslational modification. This process involves the covalent attachment of carbohydrates to specific amino acid residues, profoundly influencing protein function, folding, stability, and trafficking^1–3^. Among the various types of glycosylation, *O*-glycosylation and *N*-glycosylation are the two major categories, distinguished by the nature of the sugar–amino acid linkage^3^. In *O*-glycosylation, carbohydrates are attached to the hydroxyl groups of serine or threonine residues^4,5^. This process occurs through a series of enzymatic reactions catalyzed by glycosyltransferases, primarily within the Golgi apparatus. One key enzyme in the initiation of *O*-glycosylation is polypeptide *N*-acetylgalactosaminyltransferase 2 (GalNAc-T2), which is encoded by the GALNT2 gene^6^. GalNAc-T2 transfers *N*-acetylgalactosamine (GalNAc) to the serine or threonine residues of target proteins^7,8^, forming the Tn antigen^9,10^. The Tn antigen can be further modified by T-synthase to generate the core 1 structure, also known as the T antigen^11^. Proper folding and functionality of T-synthase require its interaction with the chaperone Cosmc in the endoplasmic reticulum^12,13^.

Alterations in *O*-glycosylation have been linked to various diseases, such as cancer, autoimmune diseases and diabetes^14^. In previous studies, we generated pancreatic acinar cell-specific *Cosmc*-knockout mice, leading to a loss of core 1 glycans^15^. *Cosmc-*deficient mice exhibit exocrine pancreatic insufficiency and diabetes, revealing an association between altered *O*-glycosylation and the pathophysiology of pancreatic disease^15^. In a subsequent study, we generated a conditional transgenic *GalNT2* mouse line to investigate the impact of GalNT2 overexpression in the pancreas in vivo^16^*.* While heterozygous overexpression resulted in a loss of acinar mass and pancreatic steatosis, homozygous overexpression caused pancreatic disintegration with a loss of pancreatic function, leading to a lethal phenotype^16^.

To better understand the impact of altered *O*-glycosylation on pancreatic function, we developed a transgenic mouse model combining homozygous *Cosmc-*knockout and heterozygous GalNT2 overexpression. This model was interbred with a pancreas-specific *Ptf1a*-Cre strain (*Ptf1a*^*cre/*+^*; Rosa26*^*GalNT2/*+^*; Cosmc*^*−/−*^), resulting in amplified expression of the Tn antigen. The model allowed us to characterize the glycosylation-dependent phenotype in the pancreas.

## Materials and methods

### Mice

The conditional *Cosmc*-knockout mouse strain has been previously described^15^. Briefly, the targeting strategy involved inserting a loxP site into intron 1 and a second loxP site, along with an FRT-flanked neomycin selection cassette, downstream of exon 2, enabling the conditional deletion of exon 2 and the entire Cosmc coding sequence. Similarly, the GalNT2 coding sequence (Gene ID: 2590) was introduced into the Rosa26 locus via homologous recombination in embryonic stem cells. A floxed (lox511-flanked) transcriptional STOP cassette was placed between the GalNT2 coding sequence and the CAG promoter to ensure Cre-dependent transgene expression^16^. Homozygous floxed *GalNT2* mice were interbred with *Ptf1a*^*Cre/*+^ mice (JAX stock #023329) to generate heterozygous (*Ptf1a*^*Cre/*+^) or homozygous (*Ptf1a*^*Cre/*+^*; Rosa26*^*GalNT2/*+^) Cre-activated offspring. All the mice were maintained on a C57BL/6J genetic background (Jackson Laboratory) and bred at the research animal facility of the University Medical Center Hamburg-Eppendorf. The mice were housed under a 12-h light/dark cycle at a controlled temperature and provided food and water ad libitum. Euthanasia was performed via CO_2_ asphyxiation followed by cervical dislocation. Genotyping was carried out via the Kappa Mouse Genotyping Hot Start Kit (PeqLab, Erlangen, Germany). The genotyping primers used were Cosmc-F 5′-CACAGAACTCACTATCCACTAGGCATGAATACAT-3′, Cosmc-R 5′-GCTCTCCCTAAATATACAACCGATTAAGAAAGTGT-3′, R26-GalNT2-F 5′-AAGACGAAAAGGGCAAGCATCTTCC-3′, R26-GalNT2-R 5′-GCAGTGAGAAGAGTACCACCATGAGTCC-3′, R26-WT-F 5′-CAATACCTTTCTGGGAGTTCTCTGC-3′, R26-WT-R 5′-CTGCATAAAACCCCAGATGACTACC-3′, Cre-F 5′-ACCAGCCAGCTATCAACTCG-3′, and Cre-R 5′-TTACATTGGTCCAGCCACC-3′. All experimental procedures were approved by the Institutional Animal Care Committee and the local animal ethical committee. All methods were performed in accordance with relevant guidelines and regulations and in accordance with ARRIVE guidelines.

### Measurement of fasting blood glucose

WT (n = 8) and GalNT2-Cosmc (n = 7) were fasted overnight (12 h), and fasting blood glucose was measured with a glucometer (Accu-Chek Aviva).

### Immunohistochemical staining

These experiments were performed as we previously described^15–17^. For immunohistochemistry, antibodies against lipase (Cel) (ab79131; Abcam), insulin (8138; CST, Beverly, MA, USA), VVA-fluorescein (FL-1231-2, Vector Laboratories) and PNA-rhodamine (RL-1072-5; Vector Laboratories) were used at a dilution of 1:100. Microscopy images were acquired using a Biorevo BZ-9000 (Keyence). Composite surface sections were generated with BZ-II Analyzer 2.1 software (Keyence). Quantification of relevant IHC areas was performed using ImageJ (version 1.54g). Relative quantification was calculated as % = (area of interest/reference area) × 100.

### Western blot

Total protein concentrations were determined via the BCA protein assay (Thermo Fisher). Samples containing 30 μg of total protein were boiled for 5 min in Laemmli buffer and separated by SDS–PAGE under reducing conditions via 4–15% Mini-PROTEAN TGX gels (Bio-Rad). Proteins were then transferred onto a nitrocellulose membrane (Thermo Fisher).

The membrane was blocked with 1× Carbo-Free Blocking Solution (Biozol) in TBS-T and incubated overnight with serum diluted 1:20 in TBS-T. Biotinylated lectins, including 10 µg/ml Vicia villosa lectin (VVA, B-1235; Vector Laboratories) and 10 µg/ml peanut agglutinin (PNA, B-1075; Vector Laboratories), were complexed with 1 µg of streptavidin-HRP (21126; Pierce, Thermo Fisher Scientific) for detection. An antibody against HSPA8 (D12F2; Cell Signaling) was used as a loading control. After five washes with TBS-T buffer, protein bands were visualized via enhanced chemiluminescence (GE Healthcare).

### RNA isolation and gene expression

Total RNA was isolated from three WT and three *GalNT2*-*Cosmc* pancreas samples via the RNeasy Mini Kit (Qiagen). The RNA was reverse-transcribed into cDNA via Transcriptor Reverse Transcriptase (Roche) with an oligo-(dT) primer following the manufacturer’s instructions. Real-time PCR was performed using Maxima SYBR Green/ROX qPCR Master Mix, 0.3 μM each forward and reverse primer, and 750 ng of cDNA per reaction on a LightCycler 480 system (Roche). The cycling conditions were 95 °C for 10 min, followed by 45 cycles of 95 °C for 15 s and 60 °C for 1 min. CT values were determined via LightCycler 480 software version 1.5 (Roche). Each sample was analyzed in duplicate, and quantitative values were normalized to GAPDH expression via the 2−ΔΔCT method. All experiments were repeated at least twice for consistency.

### Cell isolation

The mice were euthanized, and the pancreas was perfused with ice-cold PBS via the portal vein and drained by cutting the vena cava. The pancreas was excised. Murine pancreata were cut into small pieces and digested in HBSS (with Ca2+ and Mg2+) containing 10 U/ml DNase and 1 mg/ml collagenase in a shaking incubator at 37 °C for 25 min. After digestion, the pancreata were smashed and washed with PBS (1% FBS) through a cell strainer to single-cell resolution, and the pellet was collected after centrifugation at 400×*g* for 8 min. The immune cells were then enriched from the pellet via Percoll gradient centrifugation (GE Healthcare, Chicago, IL).

### Fluorescence activated cell sorting

Fc-γ receptors were blocked with a mAb (clone 2.4G2). The cells were stained with fluorochrome-conjugated Abs (CD45.2 clone 104, CD11c clone HL3; BD Pharmingen; CD11b clone M1/70, NK1.1 clone PK136, CD3 clone 17A2, CD4 clone GK1.5, CD19 clone 6D5, CD8 clone 53-6.7, CD25 clone 3C7, MHC-II clone MS/114.15.2; eBioscience, San Diego, CA). BD LSRFortessa and FACSAria (BD Biosciences, San Jose, CA) were used for cell analysis and cell sorting, respectively. The data were analyzed via FlowJo v.6.1 (Tree Star, Ashland, OR).

### Statistical analysis

Each experiment was repeated at least twice. Unless otherwise noted, the data are presented as the means ± SEMs, and a two-tailed, unpaired Student’s t test was used to compare two groups of independent samples. *P* < 0.05 was considered statistically significant. Statistical analysis was performed with Prism 9.5.1 software (GraphPad Software, La Jolla, CA, USA).

## Results

### Development of a *GalNT2*-*Cosmc* mouse model results in a smaller pancreas, reduced body weight, and a high mortality rate

**Fig. 1:**
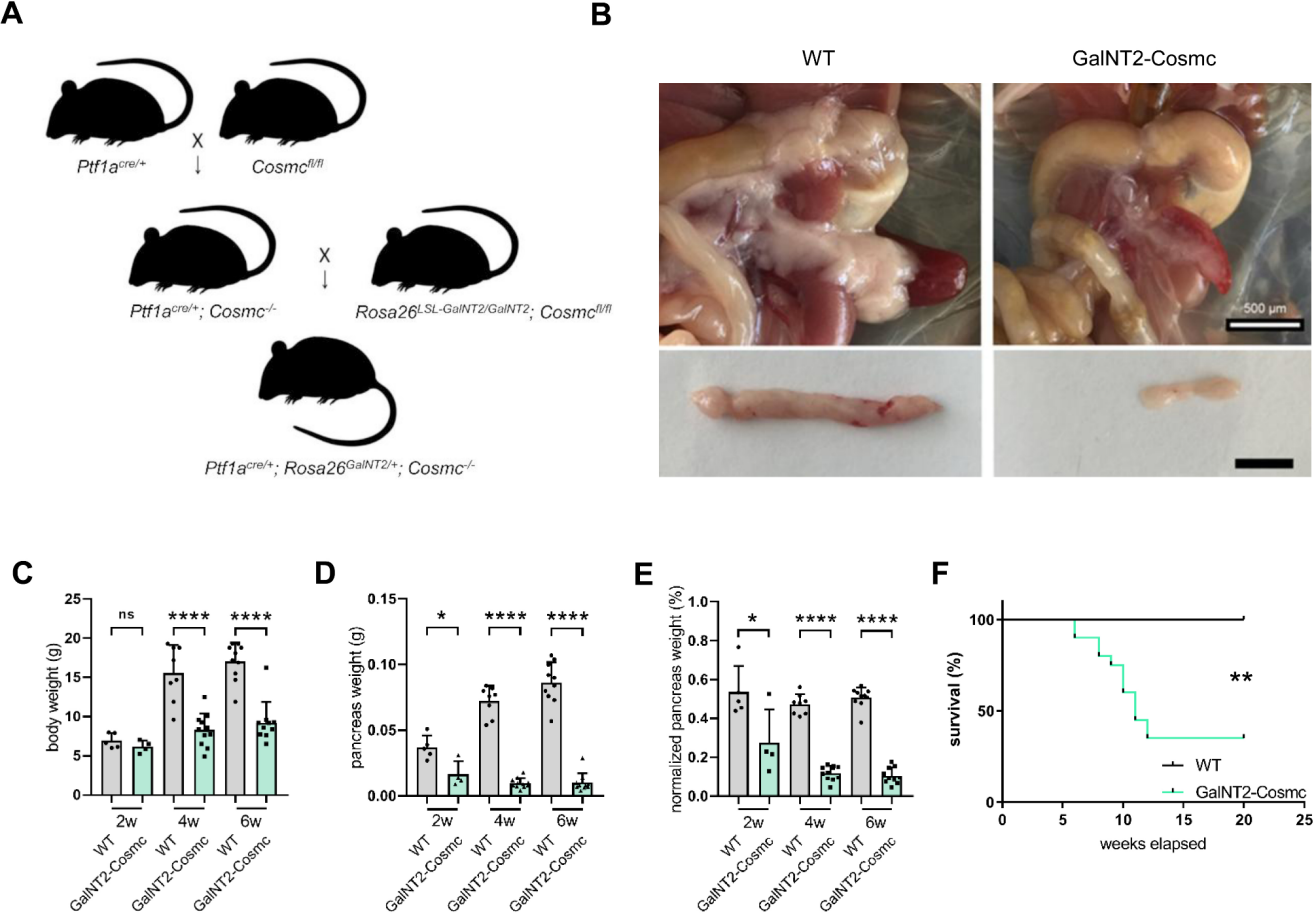
Breeding and phenotypic analysis of *GalNT2-Cosmc* mice. The starting mouse lines used to generate the *GalNT2-Cosmc* line are schematically represented in panel (**A**). Representative in situ images, along with images of excised pancreases, highlight morphological differences in the pancreas at 6 weeks of age (**B**). The scale bar represents 0.5 cm. Analysis of body weights in a cohort of wild-type (WT) and *GalNT2-Cosmc* mice at 2, 4 and 6 weeks of age revealed a significant difference at 4 and 6 weeks (*p* < 0.0001) (**C**). Similarly, examination of pancreas weights in the same cohort revealed a significant difference between WT and *GalNT2-Cosmc* mice (*p* = 0.0141 at 2 weeks; *p* < 0.0001 at 4 and 6 weeks) (**D**). Normalized pancreatic weight was reduced in *GalNT2-Cosmc* mice (*p* = 0.037 at 2 weeks; *p* < 0.0001 at 4 and 6 weeks) (**E**). The sample sizes analyzed in (**C**)–(**E**) were: 2 weeks (WT: n = 5, *GalNT2-Cosmc*: n = 4), 4 weeks (WT: n = 8, *GalNT2-Cosmc*: n = 11), and 6 weeks (WT and *GalNT2-Cosmc*: n = 10 each). A Kaplan–Meier survival analysis revealed the proportions of lethal outcomes in *GalNT2-Cosmc* mice within the first 12 weeks of life (**F**) ** *p* = 0.0016 as assessed by the log-rank (Mantel–Cox) test.

The *GalNT2-Cosmc* mouse line was established on the basis of the previously described lines *Ptf1a-*Cre*;Cosmc-*KO^15^ and *Ptf1a-*Cre*;GalNT2-*TG^16^. As shown in the breeding scheme, *Ptf1a*^*Cre/*+^*;Cosmc*^*−/−*^ mice were crossed with floxed *Cosmc*^*fl/fl*^*;Rosa26*^*LSL-GalNT2/LSL-GalNT2*^ mice to generate the genotype *Ptf1a*^*Cre/*+^*;Cosmc*^*−/−*^*;Rosa26*^*GalNT2/*+^ with the highest possible frequency (Fig. 1A). Compared with WT mice, *GalNT2-Cosmc* mice presented a noticeably smaller pancreas with a granular structure at 6 weeks of age (Fig. 1B). Analysis of body weight at 2, 4, and 6 weeks of age revealed a significant reduction in *GalNT2-Cosmc* mice compared to WT mice at 4 and 6 weeks (*p* < 0.0001), whereas no significant difference was observed at 2 weeks (Fig. 1C). Pancreatic organ weight was assessed at 2, 4, and 6 weeks of age, revealing significantly lower values in *GalNT2-Cosmc* mice compared to WT mice (*p* = 0.0141 at 2 weeks; *p* < 0.0001 at 4 and 6 weeks) (Fig. 1D). Normalized pancreatic weight was reduced in *GalNT2-Cosmc* mice (*p* = 0.037 at 2 weeks; *p* < 0.0001 at 4 and 6 weeks) (Fig. 1E). A significant proportion of *GalNT2-Cosmc* mice display a lethal phenotype (*p* = 0.0016), with a lifespan of 8–12 weeks (Fig. 1F—Kaplan–Meier).

### Progressive histological changes in the pancreas with loss of acinar tissue in *GalNT2-Cosmc* mice

**Fig. 2:**
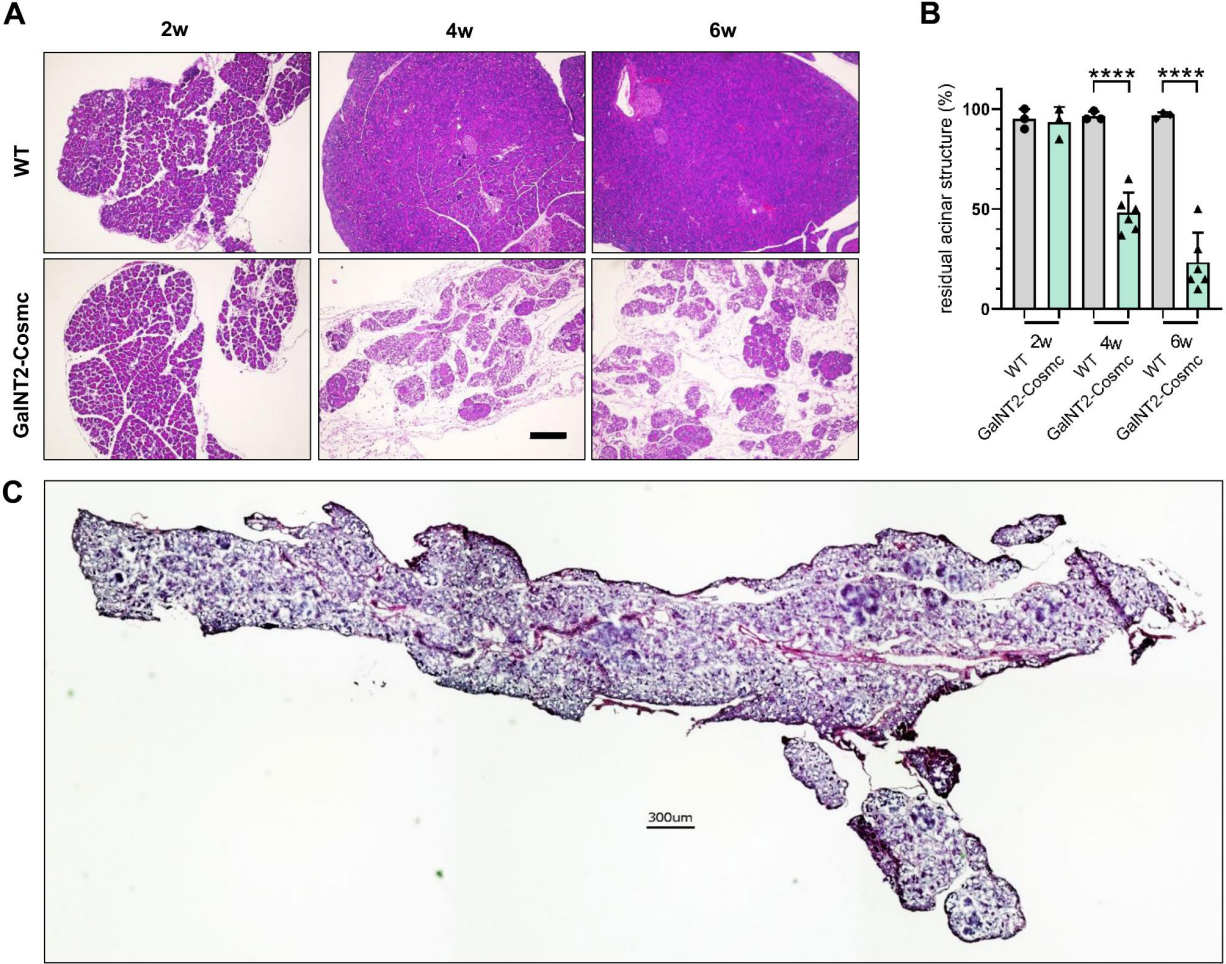
Progressive acinar tissue loss in *GalNT2-Cosmc* mice. Histological analysis revealed a progressive reduction in acinar tissue in *GalNT2-Cosmc* mice compared with WT controls. Comparative H&E staining of pancreatic sections from WT and *GalNT2-Cosmc* mice at 2, 4, and 6 weeks of age is shown (**A**). Scale bar equals 200 µm. Quantification of acinar tissue area revealed a significant reduction in *GalNT2-Cosmc* mice compared to WT mice at 4 weeks (*p* = 0.0023) and 6 weeks (*p* = 0.0020), as determined by an unpaired t-test. The number of image sections analyzed was n = 3 for WT at all ages and *GalNT2-Cosmc* at 2 weeks, and n = 6 for *GalNT2-Cosmc* at 4 and 6 weeks (**B**). A representative composite image of a *GalNT2*-*Cosmc* pancreas at 6 weeks is provided. The scale bar equals 300 µm (**C**).

H&E staining of pancreatic FFPE sections from WT and *GalNT2*-*Cosmc* mice at 2 weeks of age revealed a normally developed pancreas in both genotypes. By 4 weeks, *GalNT2-Cosmc* pancreases exhibited significant histological alterations, including dysplastic areas and marked fibrosis, which became more pronounced by 6 weeks of age (Fig. 2A). Quantification of acinar tissue revealed a significant reduction in the acinar area in *GalNT2-Cosmc* pancreases compared with WT pancreases at 4 weeks (*p* = 0.0023) and 6 weeks age (*p* = 0.0020) (Fig. 2B). A composite surface section highlights extensive tissue remodeling in *GalNT2*-*Cosmc* pancreata between 4 and 12 weeks of age (Fig. 2C).

### Histological and molecular characterization of *GalNT2-Cosmc* pancreatic tissue

**Fig. 3:**
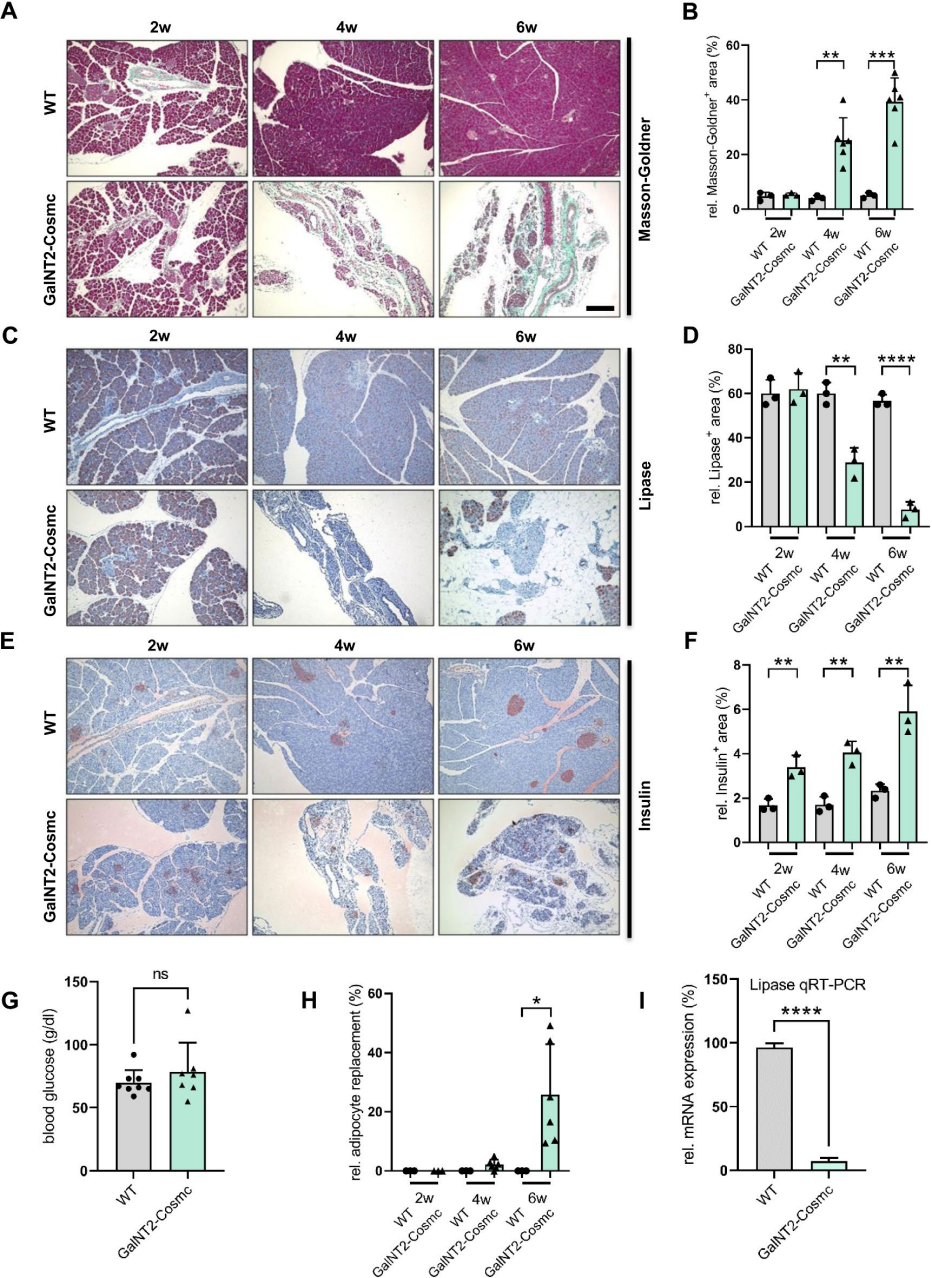
Masson–Goldner trichrome staining showing progressive collagen accumulation in *GalNT2-Cosmc* pancreases at 4 and 6 weeks. The scale bar equals 200 µm. (**A**). Relative quantification of collagen-positive areas confirmed a significant increase at 4 and 6 weeks (*p* = 0.0037 and *p* = 0.0003). The number of image sections analyzed was n = 3 for WT at all ages and *GalNT2-Cosmc* at 2 weeks, and n = 6 for *GalNT2-Cosmc* at 4 and 6 weeks (**B**). IHC staining for lipase revealed a loss of exocrine acinar cells in *GalNT2-Cosmc* pancreases from 4 to 6 weeks (**C**). Relative quantification of lipase-positive areas revealed a significant reduction at 4 and 6 weeks (*p* = 0.0029 and *p* < 0.0001). The number of image sections analyzed was n = 3 for both WT and *GalNT2-Cosmc* at all ages (**D**). IHC staining for insulin revealed a reduction in insulin-positive areas in *GalNT2-Cosmc* mice at 4 and 6 weeks (**E**). Relative quantification of insulin-positive areas, normalized to pancreatic area, showed a statistically significant increase in *GalNT2-Cosmc* mice (*p* < 0.01). The number of image sections analyzed was n = 3 for both WT and *GalNT2-Cosmc* at all ages (**F**). Measurement of fasting blood glucose in WT (n = 8) and *GalNT2-Cosmc* (n = 7) mice at 6 weeks of age showed no significant difference (**G**). Relative adipocyte replacement was observed exclusively in *GalNT2-Cosmc* mice, with measurable changes at 4 weeks of age and statistical significance at 6 weeks (*p* = 0.04) (**H**). Quantitative RT–PCR analysis revealed significant downregulation of lipase expression in the pancreas of *GalNT2*-*Cosmc* mice compared with that in WT mice (*p* < 0.0001) (**I**). All the data were statistically analyzed via an unpaired t test.

Masson–Goldner trichrome staining revealed changes in connective tissue, revealing prominent accumulation of collagen (blue/green) in *GalNT2-Cosmc* pancreases starting at 4 weeks of age, with a further increase observed at 6 weeks (Fig. 3A). The quantification of Masson-Goldner-positive areas confirmed a significant increase in collagen deposition at both 4 and 6 weeks (Fig. 3B). Immunohistochemical (IHC) staining for the exocrine marker lipase revealed a progressive loss of exocrine acinar cells in *GalNT2*-*Cosmc* pancreases from 4 to 6 weeks of age (Fig. 3C). Quantitative analysis of the lipase-positive areas revealed a significant decrease at both 4 and 6 weeks (Fig. 3D). IHC staining for insulin demonstrated that the endocrine compartment is functional in *GalNT2-Cosmc* mice at 2 weeks of age, but becomes compromised as the phenotype progresses, as evidenced by a reduction in insulin-positive areas at 4 and 6 weeks (Fig. 3E). Interestingly, relative quantification of insulin-positive areas revealed a significant increase in *GalNT2-Cosmc* mice at 4 and 6 weeks (Fig. 3F). Measurement of fasting blood glucose in WT and *GalNT2-Cosmc* mice showed no significant difference (Fig. 3G). Relative adipocyte replacement was observed exclusively in *GalNT2-Cosmc* mice, with measurable changes at 4 weeks of age and statistical significance at 6 weeks (Fig. 3H). The findings presented in Fig. 3D were further corroborated by quantitative RT–PCR analysis of pancreatic marker genes, which revealed a significant downregulation of lipase expression in *GalNT2-Cosmc* pancreases compared to WT controls (*p* < 0.0001) (Fig. 3I). Consistent with these observations, the *GalNT2-Cosmc* mice also exhibited an altered stool formation, characterized by brightened, semisolid stool in the distal colon as a consequence of exocrine pancreatic insufficiency. In contrast, the WT mice displayed fully formed pellets of stool.

### Altered glycosylation patterns in *GalNT2-Cosmc* mice with induction of Tn antigens and loss of Core 1 structures

**Fig. 4:**
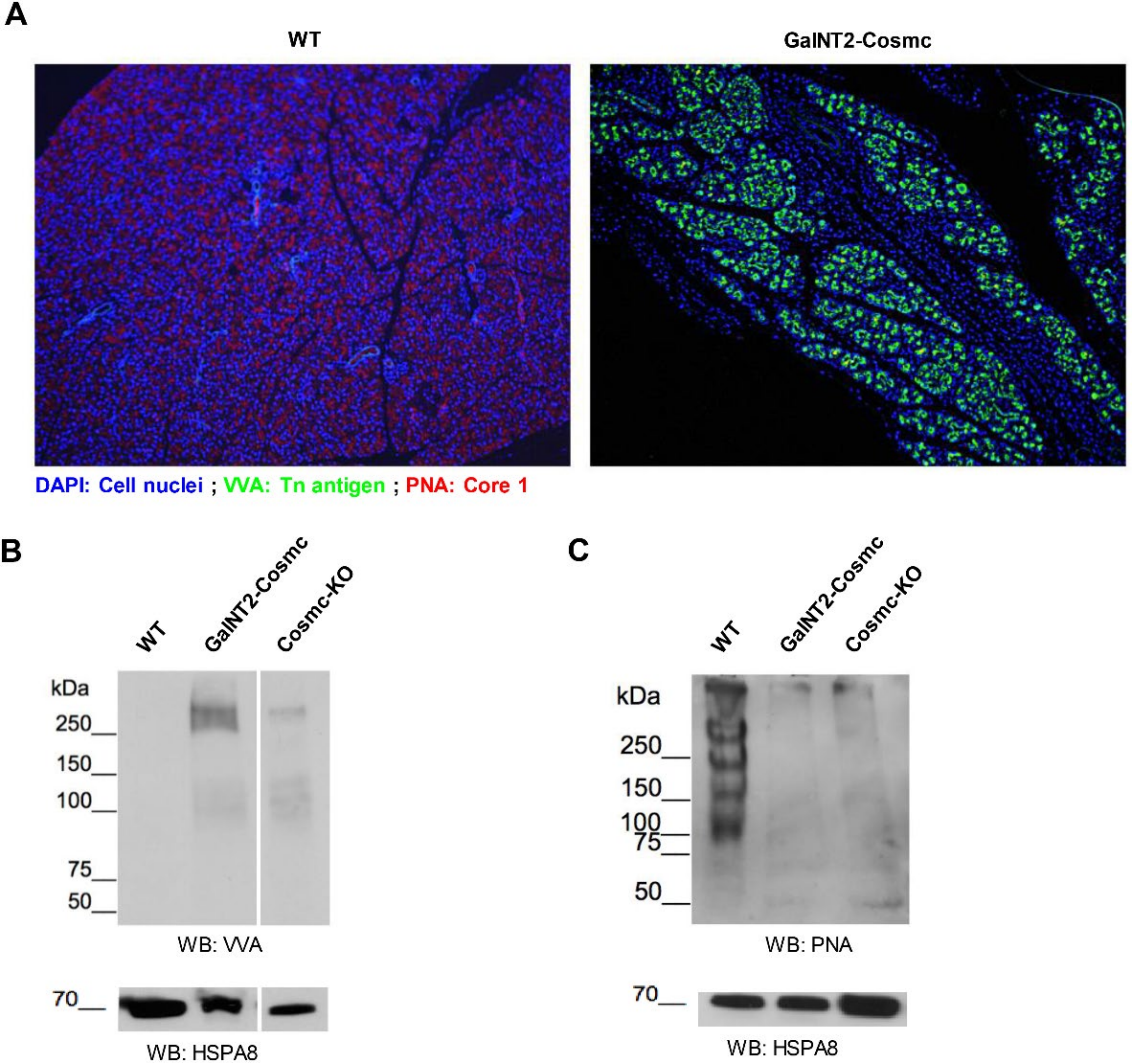
Analysis of altered glycosylation patterns in *GalNT2-Cosmc* mice via lectin staining. Fluorescently labeled lectins were employed for IF analysis of FFPE pancreatic sections from WT and *GalNT2-Cosmc* mice. PNA (red) was used to detect nonsialylated core-1 structures, whereas VVA (green) identified Tn antigens (**A**). Images were captured at 100× magnification. Western blot analysis of pancreatic lysates from WT and *GalNT2*-*Cosmc* mice was performed using the biotinylated lectins VVA and PNA, both of which were detected with streptavidin-HRP complexes (**B**,**C**). The blots shown have been cropped for clarity. Original blots are presented in Supplementary Fig. 2.

To characterize the glycosylation changes induced in the *GalNT2-Cosmc* mouse model, FFPE sections were stained with fluorescence-labeled lectins. PNA (red) was used to stain nonsialylated Core 1 structures, whereas VVA (green) was used to detect Tn antigens. The pancreas of exocrine WT mice is strongly positive for PNA, whereas the *GalNT2*-*Cosmc* genotype leads to pronounced expression of Tn antigens. The fibrotic areas showed no reactivity with either VVA or PNA (Fig. 4A). Western blot analysis of WT pancreas lysates revealed strong PNA positivity, which was nearly absent in lysates from *GalNT2-Cosmc* and *Cosmc-*KO mice (Fig. 4C). In contrast, WT pancreas lysates show no reactivity with VVA, whereas Tn antigen expression is induced in *GalNT2-Cosmc* and *Cosmc-*KO animals, resulting in VVA reactivity. A direct comparison of *GalNT2-Cosmc* and *Cosmc-*KO lysates highlights the influence of *GalNT2* overexpression on Tn antigen expression (Fig. 4B).

### Increased cell infiltration of macrophages and NK, myeloid and lymphoid DCs in the pancreas of *GalNT2-Cosmc* mice in a chronic pancreatitis model

**Fig. 5:**
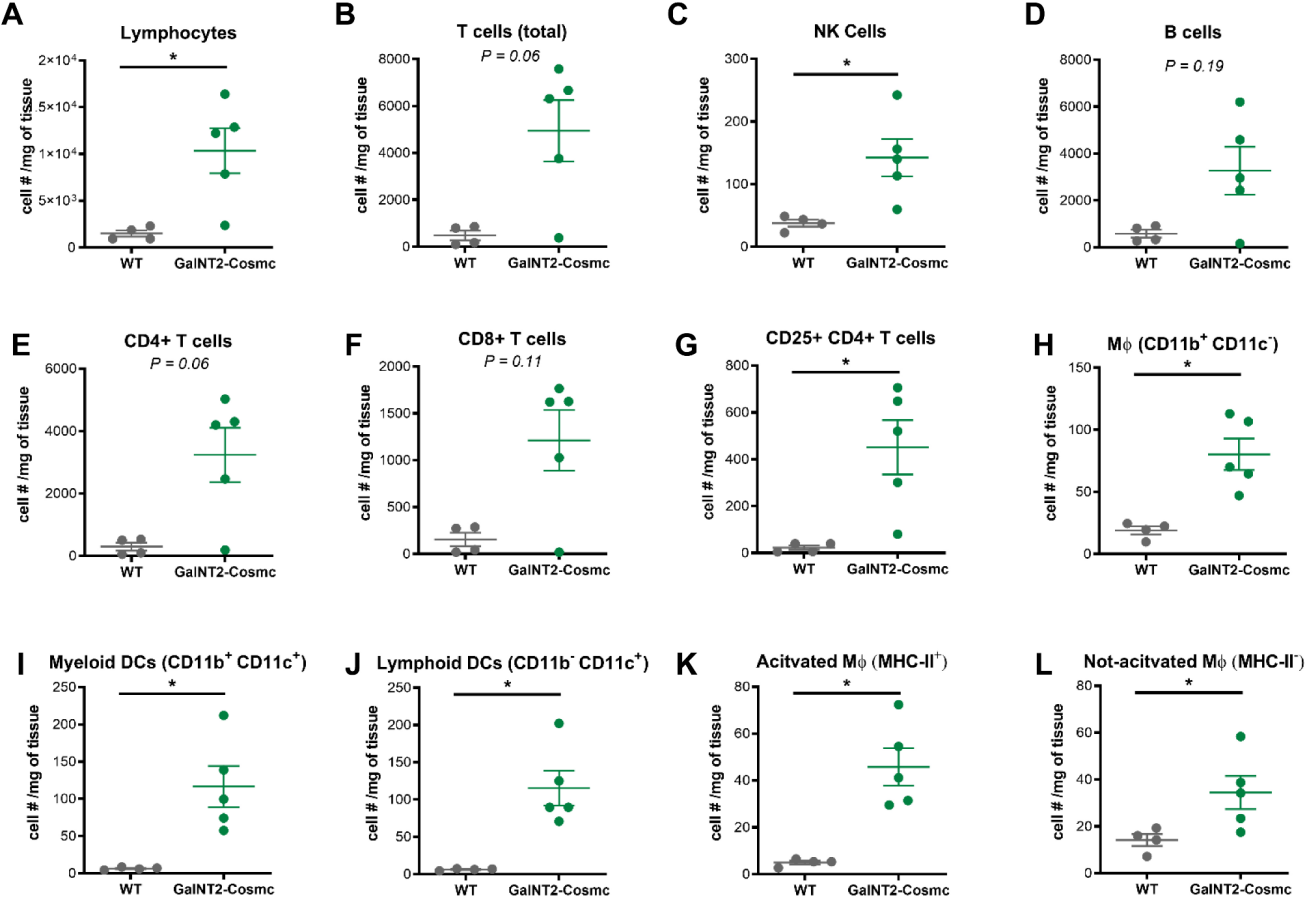
Macrophage, NK, myeloid and lymphoid DC numbers are increased in *GalNT2*-*Cosmc* mice in the chronic pancreatitis model. Flow cytometry analysis of liver-infiltrating (**A**) lymphocytes, (**B**) T, (**C**) NK, (**D**) B, (**E**) CD3+CD4+, (**F**) CD3+CD8+, (**G**) CD3+CD4+CD25+, (**H**) CD11b+CD11c−, (**I**) CD11b+CD11c+, (**J**) CD11b−CD11c+, (**K**) CD11b+CD11c−MHC-II+, and (**L**) CD11b+CD11c−MHC-II− cells in WT and *GalNT2*-*Cosmc* mice in the chronic pancreatitis model. Each dot represents one mouse. The data are presented as the means ± SEMs. The detailed gating strategy is shown in Supplementary Fig. 1. *p < 0.05, **p < 0.01 as assessed by one-way ANOVA with Bonferroni post hoc correction.

To characterize the inflammatory environment after the induction of genetic pancreatitis, we next analyzed intrapancreatic immune cell infiltration via flow cytometry. Interestingly, we detected significant increases in the absolute cell numbers of macrophages and NK cells, myeloid and lymphoid DCs in the pancreas of *GalNT2-Cosmc* mice compared with those in the pancreas of WT mice (Fig. 5A,C,G–J). Several other immune cell subsets showed a trend increase, but did not reach the statistical significance level (Fig. 5B,D–F,K,L).

**Fig. 6:**
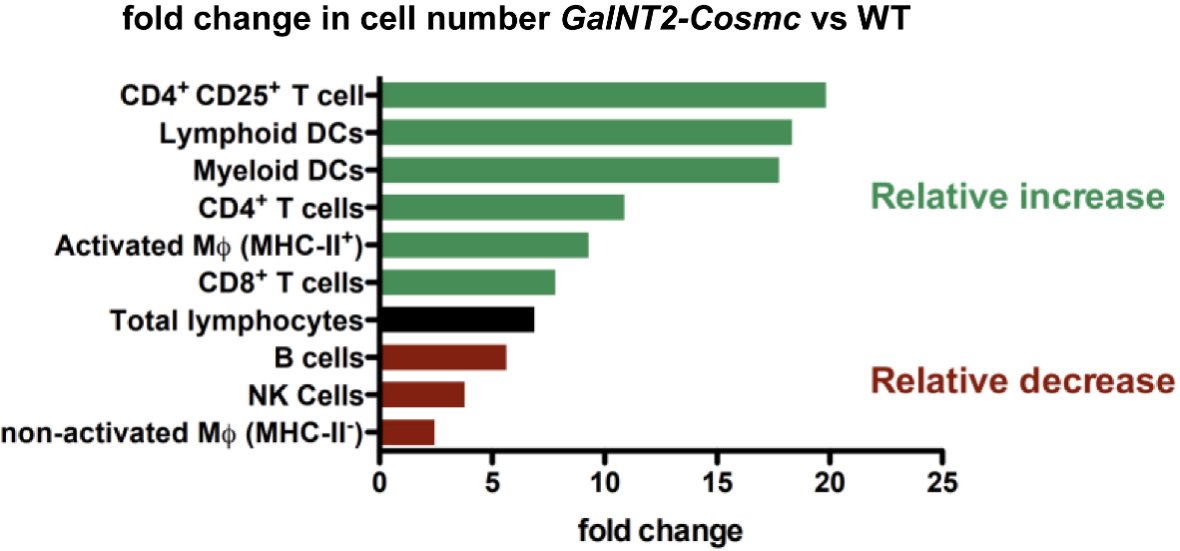
In the chronic pancreatitis model at 6 weeks of age, compared with WT mice, *GalNT2-Cosmc* mice presented a relative increase in CD4⁺CD25⁺ T cells, as well as lymphoid and myeloid dendritic cells (DCs). The fold change in immune cell numbers highlights significant differences in immune cell composition between *GalNT2-Cosmc* and WT mice under these conditions.

In conclusion, Tn antigen overexpression induces a substantial immune cell infiltration in chronic pancreatitis. Analysis of the relative abundance of immune cell subtypes further revealed an altered immune response on a cellular level in the transgenic mice. Specifically, we observed a relative increase in CD4+CD25+ T cells, lymphoid and myeloid DCs compared to the change in total lymphocyte infiltration in *GalNT2-Cosmc* mice, in comparison to those in WT mice (Fig. 6).

## Discussion

In this study, we evaluated the impact of the overexpression of the Tn antigen, a common precursor of *O*-glycans^18^, in a transgenic mouse model. Overexpression of the Tn antigen, generated by combining *Cosmc*-knockout with GalNT2 overexpression, resulted in chronic pancreatitis with acinar cell loss, fibrosis, immune cell infiltration and consecutive progressive pancreatic atrophy in the later stages. The increasing incidence of acute and chronic pancreatitis^19,20^ and the limited therapeutic options emphasize the need for a better understanding of the underlying pathophysiology. Mouse models are important and widely used to gain further knowledge about the development and pathophysiology of pancreatic diseases. The induction of pancreatitis in mouse models can be achieved pharmacologically, for example, through the frequent use of caerulein, a special diet, duct obstruction^21^ or genetic modifications. The main genetic manipulations used are knockin especially with transgenic overexpression, knockout or a combination of these two approaches^22^. Examples of commonly used models are the gain-of-function mutation in the protease serin 1 (*PRSS1*) gene^23–29^, the inactivation of *SPINK3* (pancreatic trypsin inhibitor)^30,31^, or genetic mutations in the carboxypeptidase A1 (*CPA1*) gene^32^.

To our knowledge, no previous studies have reported a link between altered *O*-glycosylation and the development of pancreatitis; however, previous studies have described the influence of impaired *O*-glycosylation on inflammation in the gastrointestinal tract. Fu et al.^10^ reported that the loss of core 1-derived *O*-glycans causes the rapid induction of severe spontaneous colitis in mice. The reduction in mucins associated with the loss of *O*-glycans in the intestine is assumed to be a major cause of inflammation^10^. Mucins are glycoproteins with large repetitive domains containing serine and threonine residues, which is why frequent GalNAc-type *O*-glycosylation is often referred to as *O*-glycosylation of the mucin type^14^. Considering the important biological role of pancreatic mucins, which create a physical barrier to protect epithelial cells, altered mucin production is assumed to be an important characteristic of inflammatory and neoplastic disorders of the pancreas^33,34^. For example, alterations in MUC1 glycosylation promote chronic inflammation, resulting in malignant transformation, and have been associated with epithelial to mesenchymal transition (EMT) and invasive growth in pancreatic cancer^2^.

In a previous study conducted by our laboratory^15^, we identified various Tn-expressing proteins in *Cosmc-*deficient mice, the majority of which were pancreatic digestive enzymes, including bile salt-activated lipase (Cel), pancreatic triacylglycerol lipase (Pnlip), pancreatic alpha-amylase 2 (Amy2) and chymotrypsin-like elastase family member 2A (Cela2a). In the literature, several genetically engineered mouse models of pancreatitis have been described that are based on autoactivation of trypsinogen^23–29^ or misfolding of other pancreatic digestive enzymes with consecutive endoplasmic reticulum (ER) stress, such as Cel^35,36^, Pnlip^37^ and Cpa1^32^. A potential contribution of Tn-overexpressing digestive enzymes to the development of chronic pancreatitis needs further investigation.

Previous studies have shown that the expression of truncated *O*-glycans resulting from incomplete *O*-glycosylation, such as the Tn antigen and its sialylated form sTn antigen, is associated with unfavorable tumor characteristics and progression in pancreatic cancer^38–40^. Thomas et al.^40^ reported that truncated *O*-glycans contribute significantly to the tumorigenesis of pancreatic cancer by inducing EMT and improving cellular plasticity and stemness. In addition, truncated *O*-glycans are involved in immunomodulation in gastrointestinal tumors^41^. The inflammatory processes of the pancreas are considered important risk factors for the development and progression of pancreatic cancer, which is also reflected in the fibroinflammatory tumor microenvironment of the pancreas^42,43^. Inflammation promotes DNA damage, resistance to apoptosis and cancer cell proliferation^44^. Inflammation of pancreatic tissue in the form of pancreatitis due to the expression of truncated *O*-glycans might therefore contribute to tumor development and progression.

In summary, we have developed a genetically engineered mouse model with hallmarks of chronic pancreatitis, including acinar cell loss, fibrosis, immune cell infiltration and progressive pancreatic atrophy, that serves as a valuable tool for investigating the molecular mechanisms underlying pancreatic diseases. This model underscores the pathophysiological importance of truncated *O*-glycan formation.

## Electronic supplementary material

Below is the link to the electronic supplementary material.

Supplementary Material 1

## Data Availability

The data are provided within the manuscript or supplementary information files.
