# Supplementary material for: Overexpression of Tn antigen induces chronic pancreatitis in mice
Source: Sci Rep. 2025 Apr 2;15:11306. doi: 10.1038/s41598-025-96060-0 (PMC11965491; doi:10.1038/s41598-025-96060-0)
Supplement: Supplementary file 1 — Supplementary Material 1 [file 41598_2025_96060_MOESM1_ESM.docx]

Supplementary Materials

**Overexpression of Tn antigen induces chronic pancreatitis in mice**

Baris Mercanoglu, Nina Schraps, Anastasios D. Giannou, Elena Neuburg, Jan Kempski, Christoph Wagener, Nathaniel Melling, Maximilian Bockhorn, Thilo Hackert and
Gerrit Wolters-Eisfeld

**This file includes:**

Supplementary Figure 1: FACS strategy for immune cell characterization.

Supplementary Figure 2: Original and uncropped blots.

**Supplementary Figure 1**

**Supplementary Fig 1. FACS strategy for immune cell characterization.**

The schematic workflow of the FACS analyses conducted is presented here. Cell analysis was performed using the BD LSRFortessa, while cell sorting was carried out with the FACSAria. Data were analyzed using FlowJo v.6.1.

**Supplementary Figure 2**

| 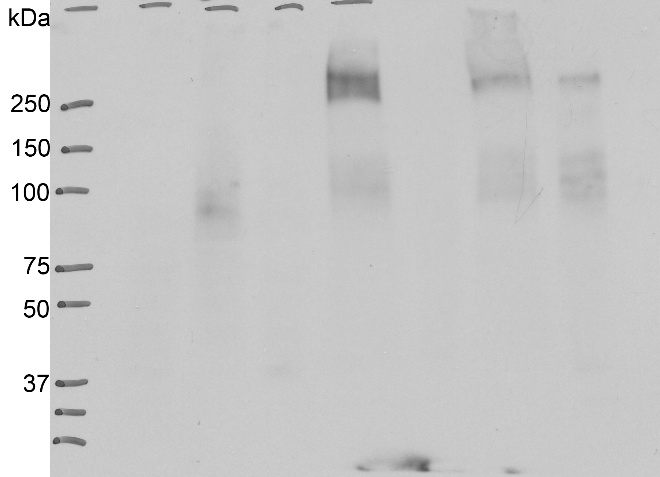 | 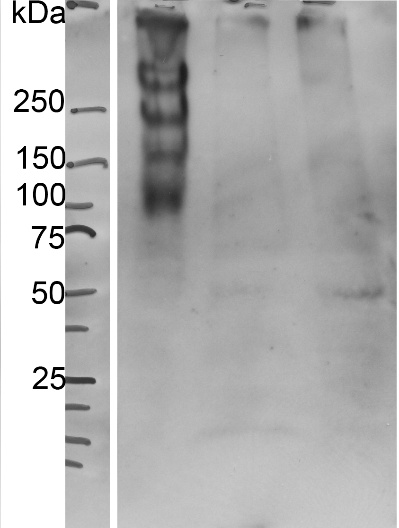 |
| --- | --- |
| WB_1: VVA | WB_2: PNA |
| 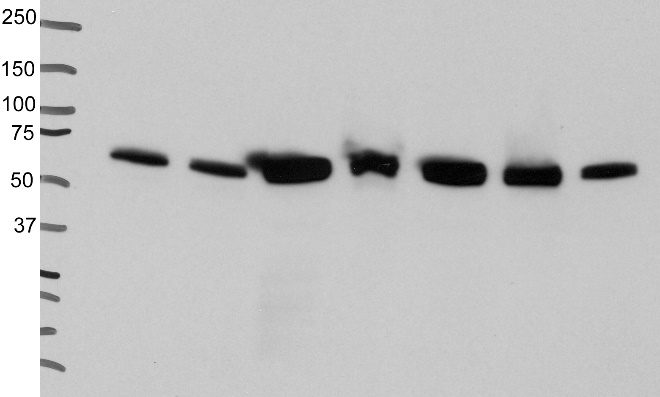 | 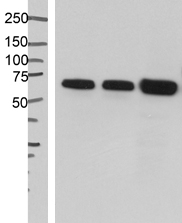 |
| WB_1: HSPA8 (~71kDa) | WB_2: HSPA8 (~71kDa) |

**Supplementary Fig 2. Original and uncropped blots.**
